# Effects of Extracellular Vesicles Derived from Human Umbilical Cord Blood Mesenchymal Stem Cells on Cell Immunity in Nonobese Mice

**DOI:** 10.1155/2024/4775285

**Published:** 2024-02-02

**Authors:** Yang Ou, Yang Yang, Yan Wang, Heng Su, Yi-kun Zhou

**Affiliations:** ^1^Department of Endocrinology and Metabolism, First People's Hospital of Yunnan Province, The Kunhua Affiliated Hospital of Kunming University of Science and Technology, Kunming 650032, China; ^2^Department of Urology, Kunming Yan'an Hospital, The Yan'an Affiliated Hospital of Kunming Medical University, Kunming 650233, China

## Abstract

Autoimmune responses are the most important pathogenic mechanisms underlying type 1 diabetes (T1D). Extracellular vesicles (EVs) derived from mesenchymal stem cells (MSCs) have immunomodulatory effects. In this study, we investigated whether EVs derived from human umbilical cord MSCs (HucMSC-EVs) have treatment effects on nonobese diabetic (NOD) mice as model of T1D. HucMSC-EVs were isolated from human umbilical cord MSCs and characterized. NOD mice (aged 4 weeks) were administered with HucMSC-EVs or the same volume of phosphate-buffered saline (PBS) via caudal vein injection twice per week. After 8 weeks of treatment, blood, spleen, and pancreatic samples were collected. Mouse blood glucose levels and body weights were measured during treatment, and insulin concentration and inflammatory cytokine levels were analyzed by enzyme-linked immunosorbent assay (ELISA). Hematoxylin and eosin (H&E) staining and immunohistochemistry (IHC) staining were used to evaluate pathological changes in mouse islets. T lymphocyte subsets were evaluated by flow cytometry, while quantitative real-time polymerase chain reaction (qRT-PCR) and Western blot (WB) analyses were used to detect the expression of transcription factor and inflammatory cytokines. Our data indicated that HucMSC-EVs treatment reduced blood glucose levels and increased insulin concentration in NOD mice. Levels of interleukin-2 (IL-2), IL-4, and IL-10 were significantly increased and those of IL-1*β* and interferon-*γ* (IFN-*γ*) significantly decreased in the HucMSC-EVs group. The positive ratio of CD4^+^ T lymphocyte subsets decreased after intravenous injection of HucMSC-EVs, in which the proportion of Th2 cells increased and that of Th1 decreased. GATA-3 and IL-2, IL-4 and IL-10 expression levels were upregulated in spleen on treatment with HucMSC-EVs, whereas those of T-bet and IFN-*γ* were downregulated. In addition, more inflammatory cell infiltration was detected in the pancreas of control group mice than those treated with HucMSC-EVs. IHC staining showed that Fas/FasL expression and distribution in control group pancreas were higher than those in the HucMSC-EVs group. Together, our findings indicate that HucMSC-EVs have potential to prevent islet injury via T cell immune responses by adjusting the Th1/Th2 ratio to regulate secretion of inflammatory factors.

## 1. Background

The main cause of type 1 diabetes (T1D) is islet infiltrated by antigen-specific autoreactive T cells and consequent autoimmune-mediated islet *β* cell destruction [1], eventually lead to a complete lack of insulin. Patients with T1D typically have a lifelong reliance on insulin, and although islet transplantation can achieve insulin independence, its clinical application is limited by rejection and donor origin. Nonobese diabetic (NOD) mice have the common features of human T1D, namely periinsulitis during the early stage of the condition and subsequent intraislet insulitis caused by lymphocyte infiltration [1].

Mesenchymal stem cells (MSCs) are pluripotent nonhematopoietic stem cells that can be extracted from a various human tissues and organs, including placenta, umbilical cord blood, pancreas, adipose tissue, and bone marrow. MSCs have been widely used in various clinical trials and animal models to regulate immune responses and protect transplanted or regenerated tissues. Further, MSCs can be used to treat T1D and its chronic complications, due to their multipotent differentiation potential and immunomodulatory function [2, 3]. Extracellular vesicles (EVs) derived from mesenchymal stem cells (MSCs-EVs) also have immunomodulatory effects and have been used as a therapy for autoimmune diseases, including T1D [4, 5]. Nakano et al. [5] found that intravenous injection of MSCs-EVs can improve cognitive impairment in a T1D model mice by repairing damaged neurons and astrocytes. Rahman et al. [1] stimulated the splenocytes of NOD mice aged 6–8 weeks with EVs released by islet-derived MSCs and found that the EVs have strong immune stimulation effects.

In this study, we explored the effects of EVs derived from human umbilical cord mesenchymal stem cells (HucMSC-EVs) on T cell immunity in T1D model mice.

## 2. Materials and Methods

### 2.1. Ethics, Consent, and Permissions

This study was carried out in accordance with the principles of Declaration of Helsinki and was approved by the Ethics Committee of the First People's Hospital of Yunnan Province. Human umbilical cord blood was collected from three healthy women. Written informed consent was obtained from all study participants. Animals were housed under standard conditions, as laid down in the National Institutes of Health of the United States guidelines regarding the care and use of laboratory animals for experimental procedures.

### 2.2. Experimental Animals

NOD/Ltf female mice (3–4 week old, Gempharmatech, Jiangsu, China) had free access to water and feed and were housed in a stable environment with a 12/12 hr light/dark cycle, 23–25°C room temperature, and 50%–60% humidity.

### 2.3. Experimental Protocol

#### 2.3.1. Animal Grouping

NOD mice (*n* = 12) were randomly divided into two groups: the control group (*n* = 6) and the HucMSC-EVs group (*n* = 6). HucMSC-EVs group animals were treated with HucMSC-EVs (2 mg/kg, dissolved in phosphate-buffered saline (PBS) to final volume of 200 *μ*l) via the caudal vein, twice a week for 8 weeks, on the third and sixth days of each week. Control group animals were injected with the same volume of PBS (200 *μ*l) on the same days.

**Figure 1:**
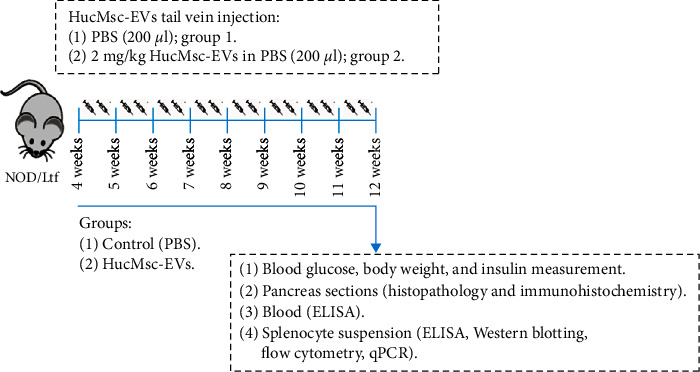
Experimental protocol. NOD mice were randomly allocated to two treatment groups. From 4 weeks old, HucMsc-EVs group mice were injected with HucMsc-EVs (2 mg/kg, dissolved in PBS to final volume of 200 *μ*l) intravenously through the caudal vein, twice a week for 8 weeks, on the third and sixth days of each week. Control animals were administered an equivalent volume of PBS on the same days.

Blood glucose and body weight of each mouse were measured weekly. At the end of the experimental procedure, all mice in both groups were dissected following cervical dislocation, and the eyeballs were removed to obtain blood. Blood samples were coagulated at room temperature for 2 hr, then centrifuged (1,000 *g*, 15 min) to separate the serum for use in enzyme-linked immunosorbent assay (ELISA) analysis. Pancreatic tissues were collected for hematoxylin and eosin (H&E) and immunohistochemistry (IHC) staining. Spleen tissues were quickly removed and used for quantitative real-time polymerase chain reaction (qRT-PCR), Western blot (WB), and preparation of single-cell suspensions for flow cytometry (Figure 1).

### 2.4. Isolation, Culture, and Characterization of HucMSC

HucMSC was isolated and identified, as previously described [6]. In brief, 1% penicillin-streptomycin double antibody (1902417, Gibco, USA) was added to the fresh anticoagulant blood samples (50 ml). After centrifugation of blood samples (700x *g*, 25 min, room temperature), there was clear stratification and the second layer, containing monocytes, was carefully aspirated into another centrifuge tube, 10 ml PBS (Sigma 5439, USA) added, and samples mixed, followed by centrifugation (250x g, 10 min). Supernatants were discarded and sediments washed twice with PBS, then resuspended in Dulbecco's modified Eagle medium supplemented with 15% (vol/vol) fetal bovine serum (FBS; Gibco, USA). Cell suspensions were placed in a gelatin-coated six-well plates and incubated with 5% CO_2_ in a humidified atmosphere at 37°C. Medium was changed after 48 hr, and nonadherent cells were removed by thoroughly washing with PBS. Colonies derived from single cells were marked, medium changed twice per week, and cells passaged using 0.25% trypsin (Gibco, USA) when they reached approximately 80%–90% confluence.

For blocking nonspecific antigens, passage 3 HucMSC was incubated with 3% bovine serum albumin for 30 min and incubated with the following monoclonal antibodies: CD34 (ab78165, Abcam), CD29 (ab36219, Abcam), CD45 (ab27287, Abcam), and CD90 (ab95700, Abcam), and analyzed by flow cytometry. Nonspecific fluorescence was determined by incubation of control cell aliquots with isotype-matched monoclonal antibodies (P8680, Solarbio, China). Cells were washed to remove unbound antibodies, and surface antigens were analyzed using a Partec GmbH CyFlow Space (Partec, Germany).

### 2.5. Isolation and Characterization of HucMSC-EVs

EVs were isolated using Ribo™ EVs Isolation Reagent (C10130-1, RIBOBIO, China) and resuspended in PBS. The specific steps are as follows: transfer the medium to serum-free culture medium. Select the culture medium supernatant, along with an appropriate amount of reagent, to a 2 ml centrifuge tube. Centrifuge at 1,500 *g* for 30 min at 4°C, discard the supernatant, and obtain a small portion of EVs. Transfer the obtained EVs to the same centrifuge tube containing 2 ml of mixed solution. Centrifuge again at 1,500 *g* for 30 min at 4°C, discard the supernatant. Repeat the above steps until all the mixed solutions have been transferred. The obtained EVs are stored at −80°C in the freezer for subsequent procedures. The identity of HucMSC-EVs was confirmed by the presence of the specific surface proteins CD9, CD63, CD81, and calnexin. The following antibodies were used: anti-CD9 (1 : 2,000, ab92726, Abcam, UK), anti-CD63 (1 : 1,000, ab134045, Abcam, UK), anti-CD81 (1 : 500, ab109201, Abcam, UK), and anti-calnexin (1 : 1,000, ab13503, Abcam, UK). Membranes were washed three times in 1x tris-HCl buffered saline Tween (TBS and 0.1% Tween 20; TBST) for 5 min, and incubated for 2 hr in TBST containing horseradish peroxidase-conjugated goat antirabbit secondary antibody (7,047, 1 : 4,000, CST). For transmission electron microscopy, EVs pellets were fixed in 2% paraformaldehyde, then loaded onto carbon-coated copper grids (AZH200, EMCN, China) with formvar mesh. Samples were incubated on grids and subsequently stained with a 2% uranyl acetate solution. Grids were examined using a transmission electron microscope (HT7700, HITACHI, Japan). Nanoparticle tracking analysis (NTA, ZetaView PMX-120-12F-R5, Particle Metrix, Germany) was used to evaluate the size distribution and concentration of nanoparticles. The protein concentration of EVs was determined using the BCA method.

### 2.6. Flow Cytometry

Flow cytometry was performed using a Partec GmbH CyFlow Space instrument, and the resulting data were analyzed using FlowJo software (TreeStar). Briefly, cells were collected by centrifugation at 2,000x *g*, then washed with blocking buffer, and stained with monoclonal antibodies for 30 min at room temperature. The following antibodies were used: CD3-FITC (MA1-80640, Invitrogen), CD3-PC5 (100218, Biolegend), CD4-PE (MCD0404, eBioscience), IFN-*γ* (11-7311-41, Invitrogen), and IL-4 (11-7042-82, Invitrogen).

### 2.7. Histopathology and Immunohistochemistry

Pancreatic tissues were fixed with 4% formaldehyde (Guanfu, China), dehydrated, and embedded using an automatic device (Leica EG1160, Germany). Histopathological observation was performed on 5 *μ*m H&E (Sigma H3136; Sigma 230251, USA) stained sections and images acquired using a Pannoramic MIDI, 3D (HISTECH, Hungary). IHC to detect Fas/FasL (Abcam, China) expression was conducted according to a standard protocol. The following antibodies were used: Fas (1 : 500, ab82419, Abcam, UK) and FasL (1 : 100, ab15285, Abcam, UK).

### 2.8. Enzyme-Linked immunosorbent Assay

Commercially available ELISA kits were used to measure serum insulin, interleukin (IL)-2, IL-4, IL-1*β*, IL-10, and interferon (INF)-*γ* (Elabscience, China); each sample was assessed in triplicate. All tests were performed in strict conformity with the manufacturer's instructions. A microplate reader (Molecular SPECTCA MAX190, USA) was used to measure optical density (OD) value. Standard curves were generated using CurveExpert software. Correlation coefficients were ≥0.999.

### 2.9. Quantitative Real-Time PCR

Total RNA was extracted from spleen tissues with Trizol Reagent (15596026, Lifetech), according to the supplier's instructions. PCR primers were designed using Beacon Designer 7.90 and synthesized by Invitrogen (China). The following were primer pairs used (forward/reverse): T-bet: 5′ TTCTATCCAACCAGTATC 3′/5′ CTGTGAGATCATATCCTT 3′; GATA-3: 5′ GAGGAGGAACGCTAATGG 3′/5′ TGCCTTCTTTCTTCATAGTCA 3′; IL−2: 5′ GATGAACTTGGACCTCTG 3′/5′ ACTCTGATATTGCTGATGAA 3′; IL−4: 5′ ATGCTTGAAGAAGAACTCTA 3′ /5′ GTGGACTTGGACTCATTC 3′; IFN-*γ*: 5′ TTAACTCAAGTGGCATAG 3′/5′ TGATTCAATGACGCTTAT 3′; IL−10: 5′ AGCAGGTGAAGAGTGATT 3′/5′ GCAGTTGATGAAGATGTCA 3′; and *β*-actin: 5′ TATGGAATCCTGTGGCATC 3′/5′ GTGTTGGCATAGAGGTCTT 3′. Reactions were conducted using the BRYT-Green detection system, with reaction volume 20 *μ*l, on the LightCycler 96 real-time PCR system (Roche, USA). Forty cycles of PCR amplification were conducted as follows: 95°C for 10 min for denaturation, 95°C for 15 s for annealing, and 60°C for 30 s for extension. *C*_T_ values were determined automatically by the real-time PCR System. Melting curves and agarose gel electrophoresis were used to confirm the specific amplification of target genes. Three technical replicates were conducted for each biological replicate. The 2^*ΔΔ*CT^ method was applied for data analysis.

### 2.10. Western Blotting

Samples were lysed with RIPA buffer and centrifuged (10 min, 1,049x *g*) to remove cell debris. Protein concentrations were determined by BCA assay (Beyotime Biotechnology, China). Equal amount of proteins (20 *µ*g) was subsequently separated by 12% SDS-PAGE and transferred to PVDF membrane, which was blocked with 5% skimmed milk and incubated with primary antibodies overnight. After washing with PBS with Tween 20 (1 hr, room temperature) and incubation overnight at 4°C with the appropriate primary antibody, membranes were incubated with secondary antibodies for 2 hr. The following antibodies were used: T-bet (1 : 1,000, ab91109, Abcam), GATA-3 (1 : 1,000, ab214804, Abcam), IL-2 (1 : 200, ab231441, Abcam), IL-4 (1 : 1,000, ab11524, Abcam), IFN-*γ* (1 : 4,000, AMC4739, Invitrogen), IL-10 (1 : 1,000, ab189392, Abcam), second antibody (1 : 2,000, CST 7074), and *β*-actin (1 : 2,000, TA-09, Zhongshan Company). Protein bands were visualized using a ChemiDoc Touch imager (Bio-Rad, USA). The gray area ratios of different bands were calculated by densitometry using ImageJ v1.8.0 software (National Institutes of Health). Each experiment was performed in duplicate.

### 2.11. Statistical Analysis

Technical replicates were performed for each experiment, with a minimum of three biological replicates for each study. Data were presented as the mean ± standard error (SEM). Statistical analyses were conducted by unpaired two-tailed *t*-test or Mann–Whitney nonparametric tests, if data did not follow a normal distribution, to determine the significance of differences among means, using a significance level of *p* < 0.05, unless otherwise specified. Data analysis was performed using GraphPad Prism Version 6.0 (GraphPad). Semiquantitative analysis of protein levels by WB was performed with ImageJ software (Bethesda, MD, USA), using *β*-actin as the loading control.

## 3. Results

### 3.1. Characterization of HucMSC

**Figure 2:**
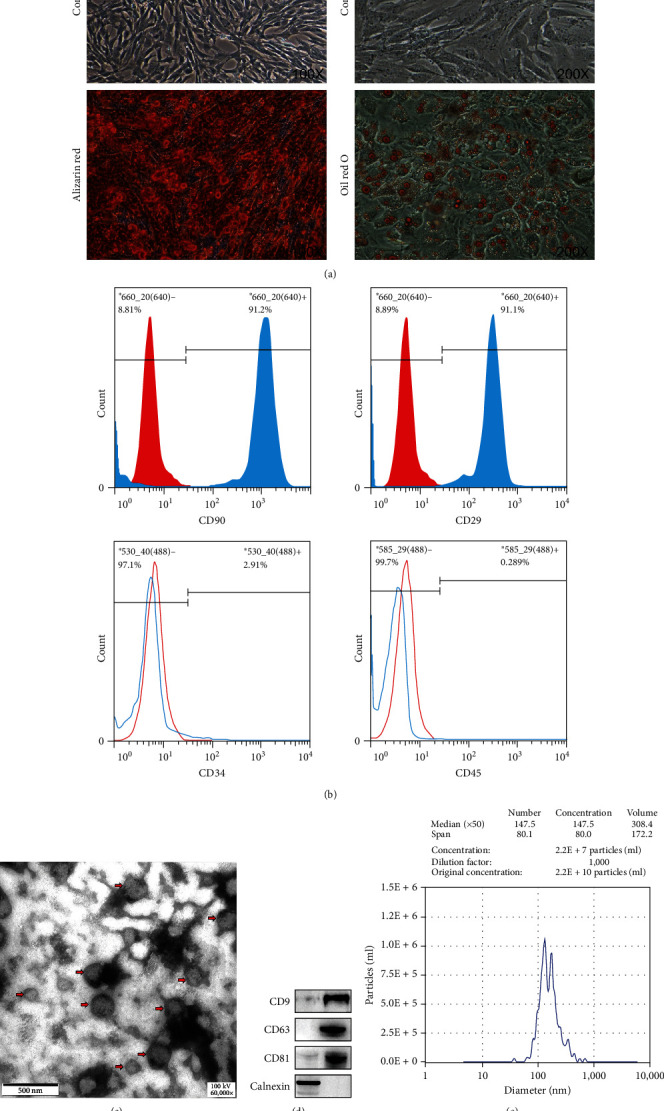
Characteristics of HucMSC and MSC-derived EVs. Adherent cells isolated from umbilical cord showed a fibroblast-like morphology. (a) Osteogenic differentiation was demonstrated by the intracytoplasmic accumulation of alkaline phosphatase. Lipid-rich drops stained with oil red O indicated adipogenesis differentiation ability. HucMSC were characterized by flow cytometry. (b) Cells were negative for CD45 and CD34, while they were positive for CD29 and CD90. (c) Morphology of MSCs-EVs under a transmission electron microscopy. (d) By Western blot analysis, specific markers for EVs (CD9, CD63, and CD81) tested positive, while calnexin tested negative. (e) NTA analysis indicated that 92.6% of MSC-derived EVs size were in the size ranged 132–175 nm, and the average concentration of HucMSC-EVs particles measured was 2.2E + 10 particles/ml.

HucMSC were separated by density gradient centrifugation and showed fibroblast-like morphology (Figure 2(a)). Cells were adherent with osteogenic vesicles, which displayed strong alkaline phosphatase activity, and numerous lipid-rich vesicles stained with oil red O, indicating adipogenic differentiation (Figure 2(a)). Flow cytometry was used to determine the immunophenotype of the human umbilical cord-derived adherent cells and showed that they were positive for CD29 and CD90 and negative for CD34 and CD45 (Figure 2(b)). Together, these results indicate that the adherent cells isolated from human umbilical cord were HucMSC.

### 3.2. Characterization of HucMSC-EVs

Next, we characterized isolated HucMSC-EVs. Transmission electron microscope and NTA demonstrated that the isolated particles contained abundant HucMSC-EVs of diameter of 132−175 nm (Figure 2(c)–2(e)). In addition, WB analysis confirmed that the EVs expressed the classical EVs-specific markers, CD9, CD63, and CD81, while not expressing calnexin. (Figure 2(d)). The concentration of EVs was determined using the BCA method and found to be 0.437 mg/ml.

### 3.3. Effects of HucMSC-EVs on Weight, Blood Glucose, and Insulin Levels

**Figure 3:**
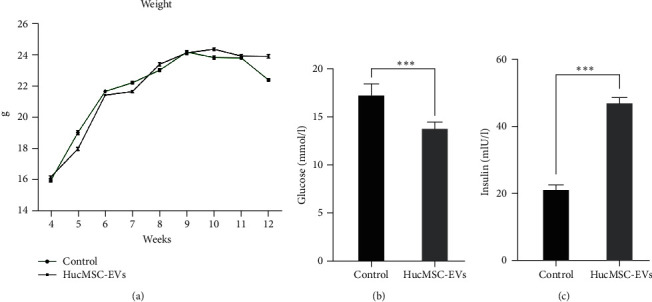
Effects of HucMSC-EVs on weight, blood glucose, and insulin levels. After 8 weeks of intravenous injection of HucMSC-EVs, (a) the weight in the control group was lower than that of HucMSC-EVs group (22.41 ± 0.21 vs. 23.92 ± 0.3 g); (b) the blood glucose level was lower than that of the control group (13.75 ± 0.29 vs. 17.22 ± 0.51 mmol/l); (c) the insulin secretion level of the control group decreased (21.19 ± 0.58 vs. 46.94 ± 0.69 mIU/l). Values are shown as the mean ± standard deviation (*n* = 6).  ^*∗∗∗*^*P* < 0.001 compared with control group.

Insulin level was detected by ELISA. After 8 weeks of intravenous injection of HucMSC-EVs, blood glucose levels were lower than those of the control group (13.75 ± 0.29 vs. 17.22 ± 0.51 mmol/l, *p* < 0.001) (Figure 3(b)). Further, insulin secretion (21.19 ± 0.58 vs. 46.94 ± 0.69 mIU/l, *p* < 0.001) (Figure 3(c)) and weight (22.41 ± 0.21 vs. 23.92 ± 0.3 g, *p* < 0.001) (Figure 3(a)) were lower in the control group than the HucMSC-EVs group. These results suggest that treatment with HucMSC-EVs can reduce blood glucose levels, increase insulin secretion, and maintain weight in NOD mice.

### 3.4. Histopathology and Immunohistochemistry

**Figure 4:**
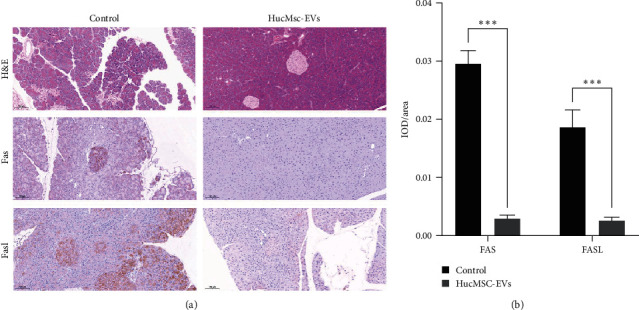
Effects of HucMSC-EVs on H&E and IHC of islets. (a) More inflammatory cell infiltration in the pancreas of the control group than hucMSC-EVs group; (b) IHC staining showed that Fas/FasL expression and distribution were higher in control group than in HucMSC-EVs group pancreas samples.  ^*∗∗∗*^*P* < 0.001 compared with control group.

Microscopic morphology (H&E staining, 10x) of islet from control and HucMSC-EVs group animals revealed more inflammatory cell infiltration in the pancreas of the control group (Figure 4(a)). IHC staining showed that Fas/FasL expression and distribution were higher in control group than in HucMSC-EVs group pancreas samples (Figure 4(b)).

### 3.5. Effects of HucMSC-EVs on Inflammatory Cytokines

**Figure 5:**
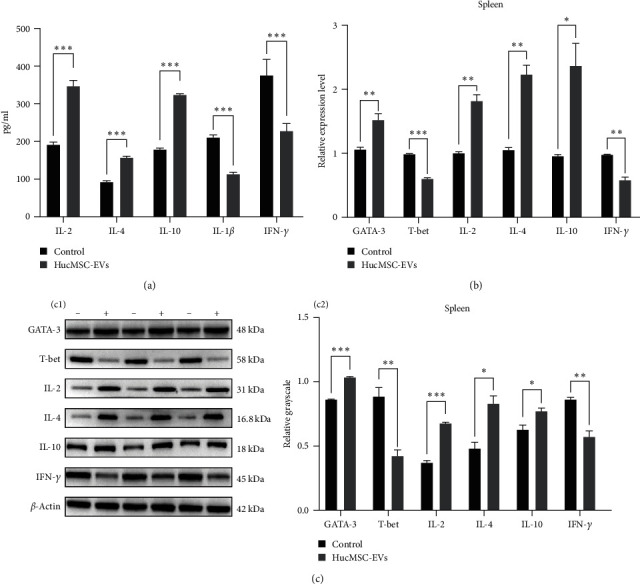
Effects of HucMSC-EVs on inflammatory cytokine and transcription factor T-bet/GATA-3. (a) HucMSC-EVs affected the level of serum inflammatory cytokines in NOD mice after intravenous injection for 8 weeks; (b) HucMSC-EVs affected the mRNA expression of spleen transcription factor T-bet/GATA-3 and inflammatory cytokines IL-2, IFN-*γ*, IL-4, and IL-10. (c(c1 and c2)) HucMSC-EVs affected the protein expression of spleen transcription factor T-bet/GATA-3 and inflammatory cytokines IL-2, IFN-*γ*, IL-4, and IL-10.  ^*∗*^*P* < 0.05,  ^*∗∗*^*P* < 0.01,  ^*∗∗∗*^*P* < 0.001 compared with control group.

Serum inflammatory cytokines, including IL-2, IL-4, and IL-10, were detected by ELISA after 8 weeks of treatment of NOD mice. Levels of IL-2, IL-4, and IL-10 were significantly higher in HucMSC-EVs group than those in the control group (346 ± 16.5 vs. 191.9 ± 6.85 pg/ml, *p* < 0.001; 156.7 ± 4.16 vs. 92.4 ± 3.58 pg/ml, *p* < 0.001; 323.5 ± 3.23 vs. 178.1 ± 5.1 pg/ml, *p* < 0.001). In contrast, IL-1*β* and IFN-*γ* levels were significantly lower in HucMSC-EVs than the control group (113.3 ± 5.28 vs. 211.1 ± 6.54 pg/ml, *p* < 0.001; 227.3 ± 20.56 vs. 375.2 ± 43.58 pg/ml, *p* < 0.001) (Figure 5(a)).

### 3.6. Effects of HucMSC-EVs on T Lymphocyte Subsets and Splenocytes Th1/Th2 Ratio

**Figure 6:**
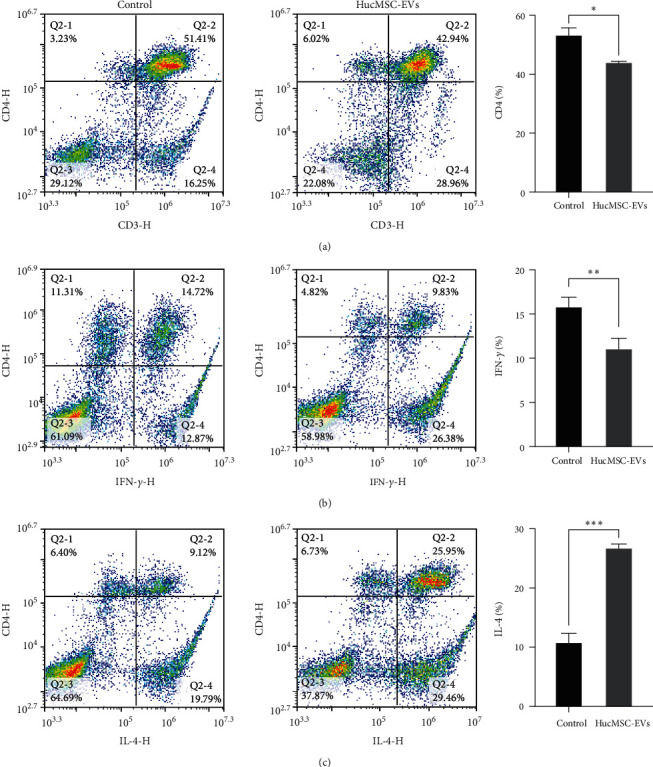
Effects of HucMSC-EVs on T lymphocyte subsets and Th1/Th2 ratio of splenocytes in NOD mice. The proportion of CD4^+^ T lymphocytes and Th1/Th2 ratio (IFN-*γ* as a marker of Th1 cells, IL-4 as a marker of Th2 cells) was detected by flow cytometry: (a) HucMSC-EVs inhibited the proliferation of CD4^+^ T lymphocyte subsets; (b) HucMSC-EVs decreased the proportion of IFN-*γ*; and (c) HucMSC-EVs increased the proportion of IL-4.  ^*∗*^*P* < 0.05,  ^*∗∗*^*P* < 0.01,  ^*∗∗∗*^*P* < 0.001 compared with control group.

Flow cytometry was used to detect CD4^+^ T lymphocyte subsets and determine the Th1/Th2 ratio, where IFN-*γ* served as a marker of Th1 cells and IL-4 as a marker of Th2 cells. After intravenous injection of HucMSC-EVs for 8 weeks, the positive ratio of CD4^+^ T lymphocyte subsets was lower than that in controls (53.17 ± 2.55 vs. 43.8 ± 0.6, *p*=0.0231) (Figure 6(a)). Further, the proportion of cells positive for IFN-*γ* was also lower in treated vs. control mice (15.77 ± 0.67 vs. 11.02 ± 0.72, *p*=0.0011) (Figure 6(b)), accompanied by a higher proportion of cells positive for IL-4 (10.72 ± 0.94 vs. 26.66 ± 0.46, *p* < 0.001) (Figure 6(c)). Hence, HucMSC-EVs could inhibit the CD4^+^ T lymphocyte subsets proliferation in NOD mice, with the Th2 proportion increased and that of Th1 cells decreased, indicating that HucMSC-EVs can influence the Th1/Th2 ratio.

### 3.7. Effects of HucMSC-EVs on the Expression of Transcription Factor T-Bet/GATA-3 and Inflammatory Cytokine in Spleen of NOD Mice

RT-PCR and WB analyses were used to evaluate the expression levels of T-bet, GATA-3, IL-2, IL-4, IL-10, and IFN-*γ*. The results showed that expression of GATA-3 transcription factor in NOD mice spleen was upregulated following intravenous injection of HucMSC-EVs for 8 weeks. By contrast, T-bet expression was downregulated. In addition, levels of the inflammatory cytokine, IFN-*γ*, were decreased in the HucMSC-EVs group, while those of IL-2, IL-4, and IL-10 were increased (Figure 5(b)). The protein expression levels of these inflammatory factors were consistent with their mRNA expression, suggesting that HucMSC-EVs affected T-bet/GATA-3 and inflammatory cytokine (IL-2, IFN-*γ*, IL-4, and IL-10) expression (Figures 5(c1) and 5(c2)).

## 4. Discussion

T1D is characterized by permanent cell destruction, resulting from autoimmune damage to islet *β* cells. These physical changes are caused by islet inflammation; that is, infiltration of islets by mononuclear cells, dominated by T cells. The etiology of T1D has yet to be clarified, but autoimmune abnormality is the main pathogenic factor [7]. In addition to autoantibodies against islets, immune cells, including CD4^+^ and CD8^+^ T lymphocytes, B lymphocytes, natural killer cells, and dendritic cells, participate in islet *β* disease by causing immune cell-mediated damage [8]. CD4^+^ T lymphocytes can recognize the N-terminal region of insulin, and mediate local inflammation or activation of apoptosis, through mechanisms involving their secreted cytokines, thereby damaging islet *β* cells [9]. CD4^+^ T cells include Th1 and Th2 cell subsets, and imbalance of Th1/Th2 cell differentiation and their cytokine products are dominant features of islet *β* cell damage during diabetes mellitus [10]. Th1 cells mainly secrete IFN-*γ*, IL-2, and TNF-*α*, while Th2 cells primarily release IL-4 and IL-10. Disruption of the immune environment balance leads to immune dysfunction, potentially including immune deficiency or autoimmune disease. Cytokines are mediators and regulators of immune responses and have important roles in T1D pathogenesis; when lymphocytes infiltrating islets produce more Th1-type than Th2-type factors, islet *β* cells are destroyed, whereas when Th2-type factors dominate, products of Th1 cells are downregulated, preventing *β* cell destruction. Hence, stimulation of Th2 cytokines, inhibition of Th1 cytokines, and regulation of the Th1/Th2 balance are of great significance for T1D prevention and treatment. In this study, NOD mice were selected as the mouse model of T1D, and 4-week-old female NOD mice were injected with HucMSC-EVs through the tail vein from 4 to 12 weeks before diabetes, which was different from previous studies. Our data show that HucMSC-EVs could prevent the progressive increase of blood glucose levels in NOD mice, delay the decline of insulin secretion, and maintain normal body weight. Further, HucMSC-EVs both altered blood cytokine levels and inhibited CD4^+^ T cell proliferation in splenocytes, while the Th2 cell proportion increased and that of Th1 cells decreased. In addition, the mRNA and protein levels of cytokines and transcription factors in splenocytes were also changed. Similarly, islet inflammation and Fas/Fasl expression were decreased following HucMSC-EVs treatment.

Several previous studies have shown potential therapeutic effects of EVs derived from MSCs on fulminant liver failure, myocardial infarction, pulmonary fibrosis, prostate cancer, obesity, T1D, and type 2 diabetes (T2D) [11–13]. Data from those studies have suggested that EVs can have opposing beneficial and harmful effects on T1D. First, the ability of EVs released by islet-derived MSCs or lymphocyte-derived to interact with immune cells, such as T and B cells, means they function as an immune system activator, which can promote pancreas *β* cell apoptosis or induce inflammatory responses, contributing to T1D pathogenesis [14, 15]. By contrast, some recent research advances have discovered new properties of EVs derived from adipose tissue-derived mesenchymal stem cells (AD-MSCs) or MSC-derived EVs, indicating that they have regulatory effects on immune cell responses and are excellent candidates for application in T1D treatment [16, 17].

In a previous study, MSCs or their EVs, when administered to diabetic mice, significantly improve hyperglycemia [18–21], reduce glycosylated hemoglobin levels [21, 22], increase insulin secretion [18, 19], and improve hepatic glucose and lipid metabolism [23]. These findings are consistent with our own results, indicating that HucMSC-EVs can effectively lower blood glucose, enhance insulin secretion, and alleviate weight loss associated with hyperglycemia in a T1D mouse model. While the overall outcomes align, the specific mechanisms explored vary. Our study primarily focuses on the impact of EVs on cellular immunity, whereas other studies have identified increased gene expression related to tissue regeneration pathways, such as Reg2, Reg3, and Amy2b, following EVs infusion. EVs derived from umbilical cord blood MSCs may alleviate insulin deficiency by activating pancreatic regeneration abilities [18]. Moreover, HucMSC-derived EVs can alleviate T2DM by reversing peripheral insulin resistance and inhibiting pancreatic *β*-cell apoptosis [19]. Intravenous administration of HucMSC-derived EVs in mice with diabetic retinopathy (DR) showed improved inflammatory response and reduced oxidative damage through the miR-17-3p-mediated targeting of signal transducer and activator of transcription 1 (STAT1) [21]. Histological analysis also demonstrated improvements in structural damage to the pancreas, kidney, and liver after injection of hUC-MSCs-sEVs [22]. In the context of T2DM rats and PA-treated L-O2 cells, EVs derived from human umbilical cord MSCs (HucMDEs) enhanced hepatic glucose and lipid metabolism by activating the AMPK pathway and inducing increased expression of autophagy-related proteins (BECN1 and MAP 1LC3B) [23].

Numerous studies have also indicated that HucMSC and HucMSC-EVs may exert their effects by influencing the proliferation of CD4^+^ T cells and related cytokines in cellular immunity. HucMSC has been shown to significantly reduce the levels of pro-inflammatory cytokines (IL-6, IL-1*β*, and TNF-*α*) in the kidneys and blood of diabetic rats [24], as well as inhibit the proliferation and differentiation of *β* cells, Th1, and Th17 cells [25]. EVs derived from HucMSC can suppress the proliferation of activated CD3/CD28 splenocytes and CD3^+^ T cells and reduce the secretion of cytokines, including IL-2, IL-6, IL-12p70, IL-22, and TNF-*α* [26]. Similarly, EVs carrying miR-146a from AD-MSCs can downregulate the expression of TNF-*α*, IL-18, and IL-1*β* [27]. Treatment with EVs secreted by adipose tissue-derived MSCs (AD-MSCs-ex) in T1D mice significantly increased the levels of IL-4, IL-10, and transforming growth factor *β*, while decreasing IL-17 and IFN-*γ* levels. Histological staining and immunohistochemical analysis confirmed that AD-MSCs-EVs treatment improved the autoimmune response in T1D mice, which was associated with a significant increase in the number of pancreatic islets [16]. MSC-EVs can also induce apoptosis in subsets of CD3^+^ and CD4^+^ cells when added to peripheral blood mononuclear cell cultures [28]. Similarly, MSC-EVs can inhibit T cell activation and development by reducing INF-*γ* production in CD4^+^ T cells and promoting the differentiation of naive CD4^+^ T cells into Th1 and Treg cells [29]. This study also found that injection of HucMSC-EVs altered both blood cytokine levels and the proliferation of CD4^+^ T cells in splenocytes, as well as changing the Th1/Th2 ratio of CD4^+^ T lymphocyte subsets. Although different studies used varying cytokine detection markers and had diverse research subjects, consistent with our research findings, they also demonstrated immunomodulatory effects on cellular immunity.

Th0 cell differentiation into Th1/Th2 cells is affected by numerous factors. T-bet and GATA-3 are the key molecules that determine the differentiation direction of Th1/Th2 cells through their transcription factors activity [30–32]. Therefore, monitoring the ratio of T-bet/GATA-3 in T1D is of great significance for the early detection, prevention and treatment of T1D, and understanding T1D pathogenesis. In our research, we found that HucMSC-EVs treatment influences T-bet/GATA-3 expression and regulates the Th1/Th2 ratio, thereby influencing inflammatory factor secretion.

This study also has some limits. We only intervened in NOD mice from 4 to 12 weeks. At present, the effect of stem cell infusion on blood glucose of NOD mice after 12 weeks is not clear. In addition, the specific targets of HucMSC-EVs infusion affecting cellular immunity need to be further exploration.

## 5. Conclusions

In conclusion, early injection of HucMSC-EVs can improve blood glucose level and insulin secretion in NOD mice, affect expression of splenocyte transcription factors, T-bet and GATA-3, adjust the Th1/Th2 ratio, and influence the secretion of inflammatory factors, thereby reducing inflammatory responses, downregulating Fas/Fasl-mediated islet *β* cell apoptosis, and finally delaying diabetes development. Therefore, early HucMSC-EVs injection has a potential therapeutic effect on diabetes, and the underlying mechanism may involve its influence on NOD mouse cellular immunity. The results of this study will help to clarify the effects of MSC-derived EVs on T cell-mediated islet *β* cell protection, which will be crucial for identification of appropriate measures to protect residual islet *β* cell function in patients with T1D and reduce the risk of chronic complications. Thus, our findings provide details of a potential novel therapy for T1D.

## Data Availability

The simulation experiment data used to support the findings of this study are available from the corresponding author upon request.

## Abbreviations

T1D: Type 1 diabetes
MSCs: Mesenchymal stem cells
HucMSC-EVs: Extracellular vesicles derived from human umbilical cord mesenchymal stem cells
ELISA: Enzyme-linked immunosorbent assay
IHC: Immunohistochemistry
H&E: Hematoxylin and eosin
qRT-PCR: Quantitative real-time PCR
IL-2: Interleukin-2
IFN-*γ*: Interferon-*γ*
NOD: Nonobese diabetic
T2D: Type 2 diabetes
FBS: Fetal bovine serum
NTA: Nanoparticle tracking analysis
AD-MSCs: Adipose tissue mesenchymal stem cells
EVs: Extracellular vesicles
PBS: Phosphate-buffered saline.
